# Surgical Treatment of Constrictive Pericarditis

**DOI:** 10.21470/1678-9741-2022-0302

**Published:** 2023

**Authors:** Brunella Bertazzo, Alejandro Cicolini, Martin Fanilla, Alejandro Bertolotti

**Affiliations:** 1 Department of Cardiac Surgery Intensive Care, Favaloro Foundation University Hospital, Buenos Aires, Argentina; 2 Department of Cardiology, Favaloro Foundation University Hospital, Buenos Aires, Argentina

**Keywords:** Constrictive Pericarditis, Pericardiectomy, Pericardial Disease, Chronic Pericarditis

## Abstract

**Introduction:**

The mainstay of the treatment of constrictive pericarditis is pericardiectomy. However, surgery is associated with high early morbidity and mortality and low long-term survival. The aim of this study is to describe our series of pericardiectomies performed over 30 years.

**Methods:**

A descriptive, observational, and retrospective analysis of all pericardiectomies performed at the Institute of Cardiology and Cardiovascular Surgery of the Favaloro Foundation was performed.

**Results:**

A total of 45 patients underwent pericardiectomy between June 1992 and June 2022, mean age was 52 years (standard deviation ± 13.9 years), and 73.3% were men. Idiopathic constrictive pericarditis was the most prevalent (46.6%). The variables significantly associated with prolonged hospitalization were preoperative advanced functional class (incidence of 38.4%, P<0.04), persistent pleural effusion (incidence of 81.8%, P<0.01), and although there was no statistical significance with the use of cardiopulmonary bypass, a trend in this association is evident (P<0.07). We found that 100% of the patients with an onset of symptoms greater than six months had a prolonged hospital stay. In-hospital mortality was 6.6%, and 30-day mortality was 8.8%. The preserved functional class is 17 times more likely to improve their symptomatology after pericardiectomy (odds ratio 17, 95% confidence interval 2.66-71; P<0.05).

**Conclusion:**

Advanced functional class at the time of pericardiectomy is the variable most strongly associated with mortality and prolonged hospitalization. Onset of the symptoms greater than six months is also a poor prognostic factor mainly associated with prolonged hospitalization; based on these data, we strongly support the recommendation of early intervention.

## INTRODUCTION

Constrictive pericarditis (CP) is a disease of the pericardium characterized by impaired ventricular diastolic filling resulting from constriction caused by a fibrotic and scarred pericardium. The typical clinical manifestation is characterized by signs and symptoms of right heart failure with preserved right ventricular (RV) and left ventricular (LV) function.

CP is due to two pathophysiological phenomena: ventricular interdependence due to confinement of the heart within a rigid pericardium and loss of transmission of breathing pressures through the pericardium, hence early LV filling is impaired. As a consequence, the volume of the right ventricle increases, and septal rebound or protodiastolic displacement of the interventricular septum occurs^[1-3]^.

Physical exam findings include jugular ingurgitation with or without Kussmaul’s sign and symptoms/signs of right heart failure. In the electrocardiogram, the most characteristic feature is a low voltage tracing. Currently, the transthoracic echocardiogram is the most accessible diagnostic tool, and diagnostic signs are pericardial thickening and notch in the interventricular septum, changes in E velocity > 25% in relation to respiratory movements, a restrictive ventricular filling pattern, reverse diastolic expiratory flow in the suprahepatic veins, and dilated inferior vein cava (IVC) without respiratory variability. On cardiac magnetic resonance imaging, pericardial thickness > 3-4 mm and real-time ventricular interdependence are diagnostic signs. Typical findings during cardiac catheterization include square root sign (dip and plateau), equalization of diastolic pressures, and ventricular interdependence assessed by a systolic area ratio > 1.1^[1]^.

The most frequent etiologies are idiopathic (IDP) and infectious/inflammatory, followed by postradiation (RDT) and postsurgical. Tuberculosis is no longer the most frequent cause, but it continues to be very prevalent in patients with human immunodeficiency virus^[1,2,4,5]^.

The mainstay of treatment of CP is pericardiectomy, class IC, according to European guidelines^[6]^. However, surgery is associated with relatively high early morbidity and mortality and low long-term survival compared to the sex- and age-matched group.

The aims of this study are to describe our series of pericardiectomies performed over 30 years and to analyze the etiologic spectrum, echocardiographic findings, as well as postoperative complications and mortality.

## METHODS

We performed a descriptive, observational, and retrospective analysis of all pericardiectomies performed at the Institute of Cardiology and Cardiovascular Surgery of the Favaloro Foundation. All patients undergoing surgical pericardiectomy for CP from June 1992 to June 2022 were included.

The etiology of CP was determined based on the patient’s history, defining as postcardiotomy (PCT) CP those patients with a history of previous cardiac or thoracic surgery, RDT CP those with a history of radiation to the thorax or mediastinum, and miscellaneous (MSL) CP those with a history of tuberculosis, purulent or bacterial pericarditis, uremic, autoimmune, or neoplastic infiltration of the pericardium, and history of thoracic trauma. Finally, IDP CP was defined as all those patients who did not fit into any previously described categorization or who had suffered at least one event of acute pericarditis during their lifetime.

Preoperative, intraoperative, and postoperative demographic characteristics were analyzed, renal failure was categorized according to the Kidney Disease Improving Global Outcome classification, and the Global Initiative for Chronic Obstructive Lung Disease classification was used for chronic obstructive pulmonary disease patients. Functional class (FC) was evaluated according to the New York Heart Association (NYHA) classification, and this variable was recategorized as preserved FC for stages I and II and advanced FC for classes III and IV.

The echocardiographic variables analyzed were: LV end-diastolic and LV end-systolic diameters in millimeters (mm), LV ejection fraction (%), RV systolic function (defined as normal when Tricuspid Annular Plane Systolic Excursion was ≥ 18 mm), diastolic relaxation pattern, transmitral E velocity (cm/s), left atrial size (defined as normal, mild, moderate, or severe enlargement according to indexed volume or area), pulmonary artery systolic pressure in mmHg, IVC defined as normal and dilated with and without inspiratory collapse, pericardial thickening, and pericardial effusion.

Within the postoperative intercurrences, vasoplegia was categorized according to the dose of noradrenaline used, being mild < 0.1 ug/kg/min, moderate between 0.1 and 0.5 ug/kg/min, and severe > 0.5 ug/kg/min. The same criteria were used to classify the severity of low cardiac output syndrome, being mild when they required a dose of dobutamine < 5 ug/kg/min, moderate between 5 and 10 ug/kg/min, and severe > 10 ug/kg/min.

In-hospital and 30-day mortalities were analyzed. For both clinical and echocardiographic follow-ups, the last visit registered at the institution was considered.

The data were analyzed with the Stata 13 program. Categorical variables were expressed as absolute value and percentage; continuous variables were expressed as mean and standard deviation (SD) or as median and interquartile range (IQR), as appropriate. The 75^th^ percentile of the hospitalization variable was used to define prolonged hospitalization being > 15 days.

Categorical variables were analyzed with Chi-square or Fisher’s test, as appropriate; continuous variables were compared with the *t*-test for those variables with normal distribution and with the Mann-Whitney U test for variables with non-Gaussian distribution. A *P*-value < 0.05 was considered significant.

## RESULTS

A total of 45 patients underwent pericardiectomy between June 1992 and June 2022, mean age was 52 years (SD +/-13.9 years), and 73.3% were male. The time from symptom onset to pericardiectomy was 365 days (IQR 354-730 days). History and clinical characteristics are described in Table 1.

**Table 1:** Demographic characteristics and preoperative comorbidities.

Variables	n=45
Sex, male	33 (73.33%)
Age, years	52 (±13.9)
Chronic renal disease	6 (13.33%)
Diabetes	2 (4.44%)
Autoimmune disease	8 (17.77%)
Arterial hypertension	7 (15.55%)
Atrial fibrillation	10 (22.22%)
COPD	7 (15.55%)
Clinical	
Asymptomatic	3 (6.67%)
Heart failure with preserved EF	39 (86.67%)
Heart failure with reduced EF	3 (6.67%)

COPD=chronic obstructive pulmonary disease; EF=ejection fraction

**Fig. 1:**
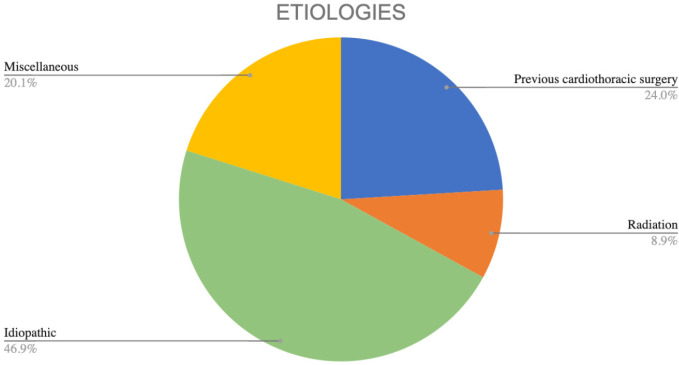
Etiologic classification of constrictive pericarditis.

Among etiologies, IDP CP was the most prevalent with 46.6%, followed by PCT CP with 23.9%. Within the latter, 50% corresponded to recurrences of CP, and the average time of recurrence was 16 years. RDT CP constituted 8.8%, and the causes of radiation were lung cancer, lymphoma, breast cancer, and a mediastinal carcinoid tumor. The average time between the application of radiotherapy and pericardiectomy was 18.5 years (IQR 11-23.5 years). MSL CP corresponded to 20%. The specifications are described in Figure 1.

Regarding the surgical approach, 71.1% were by sternotomy, and the remaining were by anterior bithoracotomy. Cardiopulmonary bypass was used in 11% of patients with a median time of 50 minutes (IQR 42-71 min). No surgery involved aortic clamping as all pericardiectomies were isolated procedures.

The preoperative and postoperative echocardiographic characteristics are described in Table 2. There is evidence of a high persistence of RV dysfunction as well as severe left atrial enlargement despite pericardiectomy. On the other hand, there was a decrease in the restrictive relaxation pattern from 33/43 (76%) patients to 6/15 (40%) patients, as well as in the dilatation of the IVC from 25/30 (83%) patients to 7/18 (38%) patients, which suggests an improvement in the overload or symptomatology. These last two variables could not be fully matched, due to the lack of information on echocardiography in the follow-up, of which only 14 studies could be matched with each other, showing a strong statistical trend (*P*=0.06) towards postoperative improvement.

**Table 2:** Comparison of preoperative and postoperative echocardiography.

Transthoracic echocardiogram	Preoperative	Postoperative
LV ejection fraction (%)^*^	60 (55-60%)	60% (55-60%)
RV dysfunction	8/45 (17.7%)	9/31 (29%)
LV end-diastolic diameter (mm)+	45.34 (± 5.2)	48 (± 5.8)
LV end-systolic diameter (mm)^*^	26 (25-29)	29 (25-39)
Left atrium		
Normal	6/42 (14.3%)	3/26 (11.54%)
Mild dilatation	16/42 (38.1%)	9/26 (34.3%)
Moderate dilatation	13/42 (30.9%)	7/26 (26.9%)
Severe dilatation	7/42 (16.7%)	7/26 (26.9%)
Restrictive LV relaxation pattern#	33/43 (76.7%)	6/15 (40%)
Pericardial thickening	33/45 (75%)	4/36 (11%)
Pericardial effusion	17/42 (40.5%)	2/39 (5.13%)
Mild	13/42 (30.9%)	
Moderate	1/42 (2.4%)	
Severe	3/42 (7.1%)	
Severe tricuspid regurgitation	3/41 (7.32%)	-
Pulmonary artery systolic pressure (mmHg)+	36.48 (± 7.11)	36.75 (± 19)
Dilated IVC without respiratory collapse#	25/30 (83.3%)	7/18 (38.8%)

* Median and interquartile range ^+^Mean and standard deviation

# Restrictive relaxation pattern and IVC of 14 echocardiograms could be paired with each other, demonstrating a statistical trend with insufficient power (*P*<0.06)

IVC=inferior vena cava; LV=left ventricular; RV=right ventricular

Among the postoperative intercurrences, atrial fibrillation represented 29.5%; acute renal failure was 13.6%, of which none required hemodialysis; and the median creatinine was 2 mg/dl (IQR 1.2-2.8 mg/dl). The incidence of postoperative pleural effusion was 46.3%, and postoperative infections were 20%. We found an incidence of vasoplegia of 33.3%, of which only two patients (13.3%) required > 0.5 ug/kg/min of noradrenaline; low cardiac output syndrome accounted for 35.5%, and there were no severe cases.

The median of days of hospitalization in the cardiovascular intensive care unit was one day (IQR 1-3 days), and length of hospital stay was eight days (IQR 5-15 days). A prolonged hospital stay (> 15 days) was observed in 26.6% of the patients. The variables significantly associated with prolonged hospitalization were preoperative advanced FC (incidence of 38.4%, *P*<0.04), persistent pleural effusion (incidence of 81.8%, *P*<0.01), and although there was no statistical significance with the use of cardiopulmonary bypass, there was a trend in this association (*P*=0.07). In addition, we found that 100% of the patients with an onset of symptoms > 6 months had a prolonged hospital stay.

In-hospital mortality was 6.6%, and 30-day mortality was 8.8% (four patients); the etiology mostly linked to mortality was RDT CP (two patients), and the other two patients corresponded to the MSL CP group, one with lymphoma/leukemia and one with purulent CP. No patient with IDP or PCT CP died. The causes of mortality in the patients who died were chronic myeloid leukemia with conversion to acute leukemia and subsequent septic shock with multiple organ failure, cardiorespiratory arrest due to asystole evolving with encephalic death, acute RV failure requiring mechanical circulatory support with extracorporeal membrane oxygenation, and finally one patient with septic shock secondary to mediastinitis/purulent CP due to *Staphylococcus aureus*.

**Fig. 2:**
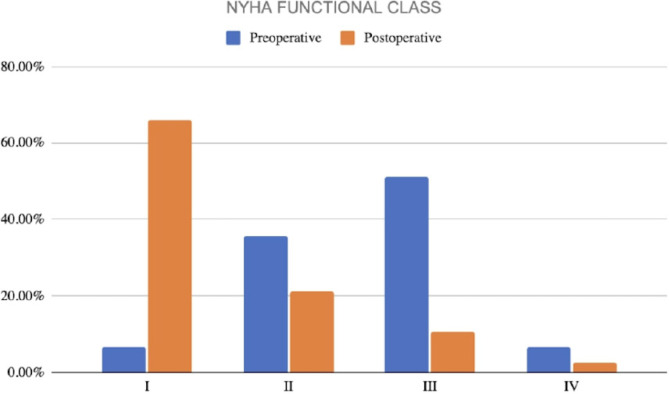
Comparison of preoperative and postoperative functional class. NYHA=New York Heart Association.

Follow-up time was 135 days (IQR 15-365 days). During follow-up, there was evidence of improvement in FC, with 65.7% of patients remaining in NYHA I and 21% in FC II (Figure 2). In the statistical analysis, we observed that preserved FC (I and II) is 17 times more likely to improve its symptomatology after pericardiectomy (odds ratio 17, 95% confidence interval 2.66-71; *P*<0.05). Only 13% of patients remained with impaired FC.

## DISCUSSION

CP and, therefore, pericardiectomies are a rare entity, so large registries are scarce; in Argentina, this analysis is the only one of its kind and has a number of patients similar to the series analyzed. Murashita et al.^[7]^ has the largest series published so far, 1,066 patients over 80 years of experience, followed by Bertog et al.^[8]^ with 166 patients.

In all the series analyzed, IDP CP is the predominant etiology, which we were able to corroborate with our analysis^[1-5,7,9-14]^. The second etiology that usually follows in prevalence is PCT CP, representing between 17 and 39% of the cases, coinciding with our report as well^[1,7,8,11,12,14]^. As for the MSL group, if we break it down, only 11% corresponds to tuberculosis. Both Porta et al.^[5]^ and Montero et al.^[4]^ in their series of 140 and 53 patients, respectively, report infectious/inflammatory CP (including tuberculosis) as second in frequency, after IDP CP. In contrast, the group of Murashita et al.^[7]^ and Porta et al.^[5]^ explain that there is a change in the etiological spectrum with a greater tendency towards PCT and RDT CP due to the decrease in tuberculosis and its complications^[4]^.

On the other hand, RDT CP is a less prevalent etiology in most series but with high morbimortality, which we were also able to prove since 50% of our mortality was due to this etiology. Several authors describe radiation as a poor prognostic factor in the development of CP and its subsequent surgical resection^[1,7,8,13]^. Bertog et al.^[8]^ describe an average of 11 years (range between 2-20 years) between exposure to radiation and pericardiectomy, in our case it was 18.5 years.

The surgical approach by median sternotomy was the most prevalent, as in most of the series compared, with an average of 70% sternotomy *vs.* 30% bithoracotomy or left anterior thoracotomy^[1,4,7,11-14]^. With their analysis over 80 years, dividing their study into two periods - “the old era” until 1990 and “the new era” since 1990 -, Murashita et al.^[7]^ reported a change in trend over time from left anterior thoracotomy to median sternotomy, due to the possibility of being able to extend the anterior pericardiectomy from phrenic to phrenic, the diaphragmatic pericardium, and, when possible, the posterior pericardium. Furthermore, these authors maintain, as do some other authors, that greater aggressiveness in the resection of the pericardium is a good long-term prognostic factor^[4,10,12,14]^. For example, Nozohoor et al.^[12]^, in their series of 41 patients, where they compare the results of radical *vs.* subtotal pericardiectomy, describe a 10-year survival rate of 94% for radical pericardiectomy, and 54% for subtotal pericardiectomy. In our experience, the trend regarding the surgical approach has changed over the last 15 years or so towards anterior bithoracotomy (Clamshell incision), which, like sternotomy, provides better bilateral and posterior access to the pericardium when the use of cardiopulmonary bypass is not necessary; the same authors recently mentioned also clarify that pericardiectomy without cardiopulmonary bypass has a better prognosis than when it is used^[4,7,12,14]^. In our analysis, cardiopulmonary bypass was used in only 11% of the cases, and we observed a strong tendency towards prolonged hospitalization in this group.

Regarding the preoperative and postoperative echocardiographic findings, the improvement of the restrictive diastolic relaxation pattern was notable in 47%, as well as the dilatation of the IVC in 54%, which strongly coincides with the improvement in FC - 65% remained in FC I and 21% in FC II after pericardiectomy. On the other hand, patients who came to surgery with RV dysfunction not only did not improve dysfunction after pericardiectomy, but there was even a 15% increase in the prevalence of such dysfunction. Both cardiac magnetic resonance and computed tomography are currently extremely useful tools, not only to confirm the diagnosis of CP, but also in the contribution with new information that escapes the echocardiogram, for example, pericardial inflammation by cardiac magnetic resonance or the evaluation of pericardial calcifications in the case of computed tomography, both methods constituting a great tool for surgical planning; in our analysis, we have very few patients who were evaluated by these techniques, but there is no doubt of their usefulness and potential uses.

Among the postoperative intercurrences, the most frequently described was low cardiac output syndrome with an incidence of 35% in our analysis, which coincides with other series^[3-5,14]^. On the other hand, the most serious postoperative complication seems to be acute renal failure requiring hemodialysis with an incidence that varies from 1.8 to 7%; as reported by several authors, renal replacement therapy significantly worsens the prognosis^[3,4,7,8,13,14]^. Our incidence of renal failure was 13%, but no patient required hemodialysis.

In our analysis, we found that in-hospital mortality was 6.6% and 30-day mortality was 8.8%. Larger series such as those by Murashita et al.^[7]^ and Bertog et al.^[8]^ describe a mortality of 5.2% and 6%, respectively. The rest of the authors with smaller series report a mortality that varies between 0 and 13%^[1,3,5,11,12,14]^. In a self-referential analysis of hospital mortality reported by Santos et al.^[9]^ in this same institution in 2008, we found a significant decrease of 50% from that date to the present.

All authors, regardless of the number of patients or mortality reported, agree that advanced FC at the time of pericardiectomy is the variable most strongly related to mortality. Although we could not demonstrate an association between mortality and FC, probably due to the limited number of patients in our study, we were able to corroborate a statistically significant association between prolonged hospitalization and preoperative advanced FC, as well as with the onset of symptoms when > 6 months, which makes us agree with authors such as Depboylu et al.^[1]^ who strongly recommend early surgical intervention due to these findings, considering early as < 6 months from the detection of CP or the onset of symptoms until the procedure.

Regarding the relationship between mortality and CP etiologies, we observed that RDT CP has the worst prognosis, since in our case it corresponds to 50% of the deaths; this association also coincides with that reported in the literature^[1,8,13]^. On the other hand, IDP CP has the best prognosis^[4,12]^. The causes of death in general are linked to low cardiac output syndrome and sepsis.

### Limitations

The main limitations of our study were its retrospective nature and its limited number of patients, and we were not able to recover all the control echocardiograms during follow-up, which limits our analysis in terms of these variables.

## CONCLUSION

IDP CP continues to be the most frequent etiology and with the best prognosis; on the other hand, RDT CP, although it has a low incidence, has high morbimortality, which is why radiation is considered a predictor of poor prognosis.

The greater aggressiveness in pericardial resection, as well as avoiding the use of cardiopulmonary bypass, means a better long-term prognosis.

Advanced FC at the time of pericardiectomy is the variable most strongly associated with mortality and prolonged hospitalization. In addition, we were able to corroborate that the onset of symptoms > 6 months is also a poor prognostic factor mainly associated with prolonged hospitalization; it is because of these data that we strongly support the recommendation of early intervention (< 6 months) in patients with less advanced FC.

Randomized and prospective studies will be necessary in the near future to confirm these assertions given the high morbimortality that still occurs in this pathology.
